# A probabilistic classifier ensemble weighting scheme based on cross-validated accuracy estimates

**DOI:** 10.1007/s10618-019-00638-y

**Published:** 2019-06-17

**Authors:** James Large, Jason Lines, Anthony Bagnall

**Affiliations:** School of Computing Sciences, University of East Anglia, Norwich, UK

**Keywords:** Classification, Heterogeneous, Ensemble, Weighted

## Abstract

Our hypothesis is that building ensembles of small sets of strong classifiers constructed with different learning algorithms is, on average, the best approach to classification for real-world problems. We propose a simple mechanism for building small heterogeneous ensembles based on exponentially weighting the probability estimates of the base classifiers with an estimate of the accuracy formed through cross-validation on the train data. We demonstrate through extensive experimentation that, given the same small set of base classifiers, this method has measurable benefits over commonly used alternative weighting, selection or meta-classifier approaches to heterogeneous ensembles. We also show how an ensemble of five well-known, fast classifiers can produce an ensemble that is not significantly worse than large homogeneous ensembles and tuned individual classifiers on datasets from the UCI archive. We provide evidence that the performance of the cross-validation accuracy weighted probabilistic ensemble (CAWPE) generalises to a completely separate set of datasets, the UCR time series classification archive, and we also demonstrate that our ensemble technique can significantly improve the state-of-the-art classifier for this problem domain. We investigate the performance in more detail, and find that the improvement is most marked in problems with smaller train sets. We perform a sensitivity analysis and an ablation study to demonstrate the robustness of the ensemble and the significant contribution of each design element of the classifier. We conclude that it is, on average, better to ensemble strong classifiers with a weighting scheme rather than perform extensive tuning and that CAWPE is a sensible starting point for combining classifiers.

## Introduction

Investigation into the properties and characteristics of classification algorithms forms a significant component of all research in machine learning. Broadly speaking, there are three families of algorithms that could claim to be state of the art: support vector machines; multilayer perceptrons/deep learning; and tree based ensembles. Nevertheless, there are still good reasons, such as scalability and interpretability, to use simpler classifiers such as decision trees. Thousands of publications have considered variants of these algorithms on a huge range of problems and scenarios. Sophisticated theories into performance under idealised conditions have been developed and tailored models for specific domains have achieved impressive results. However, data mining is an intrinsically practical exercise and our interest is in answering the following question: if we have a new classification problem or set of problems, what family of models should we use given our computational constraints? This interest has arisen from our work in the domain of time series classification (Bagnall et al. 2017) and through working with many industrial partners, but we cannot find an acceptable answer in the literature. Large-scale comparative studies of classifiers attempt to give some indication (e.g, Fernández-Delgado et al. 2014), but most people make the decision for pragmatic or dogmatic reasons.

Our first hypothesis is that, in the absence of specific domain knowledge, it is in fact better to ensemble classifiers from different families rather than intensify computational efforts into selecting and optimising a specific type. Our second hypothesis is that the best way of combining a small number of effective classifiers is to combine their probability outputs, weighted by an accuracy estimate derived through cross-validation on the training data, raised to the power four to magnify differences in competence. We call this weighting scheme the cross-validation accuracy weighted probabilistic ensemble (CAWPE). The algorithm has the benefit of being very simple and easy to implement, trivially parallelisable, incremental (in that new classifiers can be added to the ensemble in constant time) and, on average, provides state-of-the-art performance. We support the last claim with a series of experiments on two data archives containing over 200 datasets using over twenty different classification algorithms. We compare classifiers on unseen data based on the quality of the decision rule (using classification error and balanced classification error to account for class imbalance), the ability to rank cases (with the area under the receiver operator characteristic curve) and the probability estimates (using negative log likelihood).

The algorithms we compare against can be grouped into three classes: heterogeneous ensembles; homogeneous ensembles; and tuned classifiers. The first of these classes is in direct competition with our approach, while the latter two are examples of attempts to improve individual types of classifiers.

The heterogeneous ensemble algorithms most similar to our approach involve alternative weighting schemes (Kuncheva and Rodríguez 2014), ensemble selection algorithms (Caruana and Niculescu-Mizil 2004) and stacking techniques (Džeroski and Ženko 2004). We compare CAWPE to nine variants of these heterogeneous ensembles that all use the same base classifiers and the same estimate of accuracy found through train set cross-validation. We demonstrate that CAWPE provides a small, but significant, improvement on all of them.

To put the performance of CAWPE in a wider context we also compare it to homogeneous ensembles and tuned single classifiers. We choose classifiers to compare against from among those often considered to be state of the art: random forest; support vector machines; neural networks; and boosting forests. Using data derived from the UCI archive, we find that a small ensemble of five untuned simple classifiers (logistic regression, C4.5, linear support vector machine, nearest neighbour classifier and a single hidden layer perceptron) combined using CAWPE is not significantly worse than either state-of-the-art untuned homogeneous ensembles, nor tuned random forest, support vector machine, multilayer perceptron and gradient boosting classifiers.

To avoid and correct for any danger of dataset bias, we repeat the core experiments on a completely separate repository, the UCR archive of time series classification problems (Bagnall et al. 2018), and draw the same conclusions. We show that the CAWPE scheme can provide a small, but significant, improvement to the current state-of-the-art time series classification algorithm.

We then address the question as to why CAWPE does so well. We compare CAWPE to choosing the best classifier and find that the CAWPE approach is significantly better. It is most effective for data with small train set size. CAWPE consists of four key design components: using heterogeneous classifiers; combining probability estimates instead of predictions; weighting these probabilities by an estimate of the quality of the classifier found on the train data; and increasing the differences of these weights by raising them to the power $\alpha$, the single parameter of the classifier. On their own, none of these components are novel. Our contribution is to demonstrate that when used together, the whole is greater than the sum of the parts. To demonstrate this we perform an ablation study for the last three design components of CAWPE and show that each element contributes to the improved performance. We perform a sensitivity analysis for the parameter $\alpha$ and show that CAWPE is robust to changes to this parameter, but that the default value of $\alpha =4$ we decided on a priori and use in all experiments may be improved with tuning. The exponentiation through the parameter $\alpha$ allows for the amplification of small differences in accuracy estimates. This facilitates base classifiers that show a clear affinity to a given problem to provide a larger contribution to the ensemble while still allowing it to be overruled when enough of the other base classifiers disagree. It provides a mechanism to balance exploiting information found from the train data (through high $\alpha$) and mitigating for potential variance in the accuracy estimate (through lower $\alpha$).

In summary, the remainder of this paper is structured as follows. Section 2 provides a brief background into ensemble classifiers, concentrating on the algorithms most similar to CAWPE. Section 3 describes the CAWPE classifier and motivates the design decisions made in its definition. Section 4 describes our experimental design, the datasets used, and the evaluation procedure. Section 5 contains our assessment of the CAWPE classifier. We compare CAWPE to its components (Sect. 5.1), other heterogeneous ensemble schemes (Sect. 5.2), homogeneous ensemble schemes (Sect. 5.3), and tuned state-of-the-art classifiers (Sect. 5.4) on 121 UCI datasets. We also present a reproduction study of the performance gain between CAWPE and its base classifiers on the UCR time series classification datasets (Sect. 5.5), and compares its performance to the standard benchmark classifier in that domain. Section 6 provides a deeper analysis into the CAWPE scheme. We explore the differences in performance between combining a set of classifiers with CAWPE and picking the best of them based on the train set of any given dataset (Sect. 6.1). To better understand the nature of the improvements, we also carry out an ablation study that builds up from simple majority voting to CAWPE (Sect. 6.2), and perform a sensitivity analysis of CAWPE’s parameter, $\alpha$ (Sect. 6.3). Finally, we conclude in Sect. 7. Our conclusion is that it is, on average, better to ensemble the probability estimates of strong classifiers with a weighting scheme based on cross-validated estimates of accuracy than expend resources on a large amount of tuning of a single classifier and that the CAWPE scheme means that classifiers can be incrementally added to the ensemble with very little extra computational cost.

## Background

We use the following notation. A dataset *D* of size *n* is a set of attribute vectors with an associated observation of a class variable (the response), $D=\{(\varvec{x_1},y_1),\ldots ,(\varvec{x_n},y_n)\}$, where the class variable has *c* possible values, $y \in \{1,\ldots ,c\}$ and we assume there are *m* attributes, $\varvec{x_i}=\{x_{i,1},\ldots ,x_{i,m}\}$. A learning algorithm *L*, takes a training dataset $D_r$ and constructs a classifier or model *M*. To avoid any ambiguity, we stress that all model selection, parameter tuning and/or model fitting that may occur with any classifier are conducted on the train set, which may or may not require nested cross-validation. The final model *M* produced by *L* by training on $D_r$ is evaluated on a test dataset $D_{e}$. A classifier *M* is a mapping from the space of possible attribute vectors to the space of possible probability distributions over the *c* valid values of the class variable, $M(\varvec{x})=\varvec{\hat{p}}$, where $\varvec{\hat{p}}=\{\hat{p}(y=1|M,\varvec{x}),\ldots ,\hat{p}(y=c|M,\varvec{x})\}$. Given $\hat{\mathbf{p}}$, the estimate of the response is simply the value with the maximum probability.

$$\begin{aligned} \hat{y}={{\,\mathrm{arg\,max}\,}}_{i\in \{1,\ldots ,c\}} \hat{p}(y=i|M,\varvec{x}). \end{aligned}$$

An ensemble *E* is a collection of classifiers $\varvec{E}=\{M_1, \ldots , M_k\}$ built by a set of (possibly identical) learning algorithms $\varvec{L}=\{L_1, \ldots , L_k\}$ which train on (possibly different) train data $\varvec{D}=\{D_1, \ldots , D_k\}$. An ensemble algorithm involves defining the learning algorithms $\varvec{L}$, the data $\varvec{D}$ used by each learning algorithm to produce the models $\varvec{E}$ and a mechanism for combining the output of the *k* models for a new case into a single probability distribution or a single prediction.

Key concepts in ensemble design are the necessity to inject diversity into the ensemble (Dietterich 2000; Opitz and Maclin 1999; Geurts et al. 2006; Hansen and Salamo 1990) and how to combine the outputs of the models, be that through some form of voting scheme (Kuncheva and Rodríguez 2014) or meta-classification (Wolpert 1992). An ensemble needs to have classifiers that are good at estimating the response in areas of the attribute space that do not overlap too much. That being said, there is no single precise definition or measure of diversity accepted throughout the literature, with dozens of different candidates having been proposed (Kuncheva and Whitaker 2003; Tang et al. 2006). Further, it has been argued that diversity is a necessary but not itself sufficient condition of a strong ensemble (Didaci and Roli 2013), with conditions of minimal performance of the base classifiers and suitable combination methods playing a role. Broadly speaking, diversity can be engineered by either changing the training data or training scheme for each of a set of the same base classifier to form a homogeneous ensemble or by employing different classification algorithms to train each base classifier, forming a heterogeneous ensemble.

### Heterogeneous ensembles

Heterogeneous ensemble design focuses on how to use the output of the base classifiers to form a prediction for a new case. i.e., given *k* predictions $\{\hat{y_1}, \ldots , \hat{y_k}\}$ or *k* probability distributions $\{\varvec{\hat{p_1}},\ldots , \varvec{\hat{p_k}} \}$, how to produce a single prediction $\hat{y}$ or probability distribution $\varvec{\hat{p}}$. There are three core approaches: define a weighting function on the model output (weighting schemes); select a subset of the models and ignore other output (ensemble selection schemes); or build a model on the training output of the models (stacking) (Re and Valentini 2011).

#### Weighting schemes

The family of techniques most similar to our approach are weighted combination schemes, which estimate a weight $w_j$ for each base classifier and then apply it to their predictions. Base classifier predictions multiplied by some weight are summed,

$$\begin{aligned} s_i =\sum _{j=1}^{k} w_j\cdot d(i,\hat{y_j}) \end{aligned}$$

where

$$\begin{aligned} d(a,b)={\left\{ \begin{array}{ll} 1, &{} \text {if } a == b\\ 0, &{} \text {otherwise} \end{array}\right. } \end{aligned}$$

then the class with the highest weighted prediction is chosen

$$\begin{aligned} \hat{y}={{\,\mathrm{arg\,max}\,}}_{i\in \{1,\ldots ,c\}} s_i. \end{aligned}$$

Based on the framework described by Kuncheva and Rodríguez (2014), we concentrate on four weighting schemes, which are described as following on from one another when relaxing assumptions about base classifiers’ performance.

1. Majority vote (MV): $w_j=1$ for all base classifiers.
2. Weighted majority vote (WMV): $w_j$ is set as an estimate of the accuracy of the base classifier found on the train data.
3. Recall (RC): Rather than a single weight $w_j$, a separate weight is assigned to each class $w_{i,j}$. This weight is set to be the proportion of cases correct for that class on the training data (the true positive rate/recall/sensitivity).
4. Naive Bayes combiner (NBC): The Naive Bayes combiner uses the conditional distributions to form an overall distribution, assuming conditional independence.
  $$\begin{aligned} \hat{p}(y=i|\{\hat{y_1}, \ldots , \hat{y_k}\})=\hat{p}(y=i|\hat{y_1})\cdot \hat{p}(y=i|\hat{y_2}), \ldots , \hat{p}(y=i|\hat{y_k}) \end{aligned}$$
  where the probability estimates are derived directly from the train cross-validation confusion matrix. The final prediction is the index of the maximum probability.

#### Ensemble selection

A popular approach is to use a heuristic to select a subset of classifiers. Also referred to as an overproduce and choose strategy or ensemble pruning, it was initially proposed for ensembles of diverse neural networks (Partridge and Yates 1996), but later became generalised to other classifier types (Giacinto and Roli 2001). The approach became known to a wider audience after a landmark paper by Caruana and Niculescu-Mizil (2004), which describes the algorithm we implement and call ensemble selection (ES).

Given a set of base classifiers, ES uses forward selection to progressively build the ensemble, selecting the classifier at each stage that gives the largest improvement to the ensemble’s performance, or stopping when no improvement can be made. This process has a large potential for overfitting, and so this is mitigated through three strategies: selecting with replacement allows for the incorporation of good models multiple times, instead of being forced to select poor models sooner that may by chance improve ensemble performance on the current set; initialising the ensemble with a subset of the best classifiers in the pool gives a strong and reasonable start to the process; and lastly, repeating the selection process multiple times on bagged subsamples of the set of base classifiers before aggregating into a final ensemble gives the inter-relationships between different sets of models more chances to be recognised.

#### Stacking

The third popular approach to building heterogeneous ensembles is stacking (Wolpert 1992). This involves taking the output of the base classifiers on the train data, then applying another learning algorithm to determine how to best combine the outputs to predict the class value. Thus the cross-validation on the train data produces a set of predictions or probabilities for each case from all ensemble members and a further classifier is then trained on this output. New cases are classified by first producing the output of the base classifiers, then passing these outputs to the meta-classifier to form a prediction. The first stacking algorithm to gain widespread usage was stacking with multi-response linear regression (SMLR) (Ting and Witten 1999). Two extensions to SMLR were proposed by Džeroski and Ženko (2004). These were stacking with multi-response linear regression on extended features (SMLRE) and stacking with multi-response model trees (SMM5).

### Homogeneous ensembles

Homogeneous ensemble design focuses more on how to diversify the base classifiers than on how to combine outputs. Popular homogeneous ensemble algorithms based on sampling cases or attributes include: Bagging decision trees (Breiman 1996); Random Committee, a technique that creates diversity through randomising the base classifiers, which are a form of random tree; Dagging (Ting and Witten 1997); Random Forest (Breiman 2001), which combines bootstrap sampling with random attribute selection to construct a collection of unpruned trees; and Rotation Forest (Rodriguez et al. 2006), which involves partitioning the attribute space then transforming in to the principal components space. Of these, we think it fair to say Random Forest is by far the most popular. These methods combine outputs through a majority vote scheme, which assigns an equal weight to the output of each model.

Boosting ensemble algorithms seek diversity through iteratively re-weighting the training cases and are also very popular. These include AdaBoost (Adaptive Boosting) (Freund and Schapire 1996), which iteratively re-weights based on the training error of the base classifier; Multiboost (Webb 2000), a combination of a boosting strategy (similar to AdaBoost) and Wagging, a Poisson weighted form of Bagging; LogitBoost (Friedman et al. 1998) which employs a form of additive logistic regression; and gradient boosting algorithms (Friedman 2001), which have become popular through the performance of recent incarnations such as XGBoost (Chen 2016). Boosting algorithms also produce a weighting for each classifier in addition to iteratively re-weighting instances. This weight is usually derived from the the training process of the base classifier, which may involve regularisation if cross-validation is not used.

## The cross-validation accuracy weighted probabilistic ensemble (CAWPE)

The key features that define the weighting scheme we propose in the context of other commonly used weighting schemes such as those described above are that, firstly, we weight with accuracy estimated through cross-validation instead of a single hold-out validation set, secondly, we extenuate differences in accuracy estimates by raising each estimate to the power of $\alpha$ and thirdly, we weight the probability outputs of the base classifiers instead of the predictions. To clarify, prediction weighting takes just the prediction from each member classifier,

$$\begin{aligned} \hat{p}(y=i|{\varvec{E}},\varvec{x}) \propto \sum _{j=1}^k w_j d(i,\hat{y_j}) \end{aligned}$$

whereas probability weighting weights the distribution each classifier produces,

1 $$\begin{aligned} \hat{p}(y=i|{\varvec{E}},\varvec{x}) \propto \sum _{j=1}^k w_j \hat{p}_j(y=i|M_j,\varvec{x}). \end{aligned}$$

**Fig. 1:**
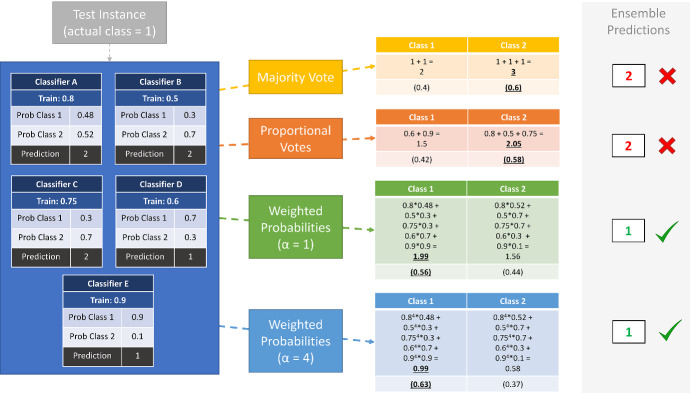
Illustration of the different effects of combination and weighting schemes on a toy instance classification. Each stage progressively pushes the predicted class probabilities further in the correct direction for this prediction

Figure 1 gives an overview of the components of CAWPE that make it different to majority voting.

Our approach is based on the idea of building a smaller number of effective classifiers and combining the output rather than learning a huge number of weak classifiers. The rationale for using the probability estimates rather than the predictions is that they will contain more information than a point estimate, and with fewer classifiers we need to capture all information available. With 500 base classifiers the voting mechanism is less important than with 5 classifiers, since averaging over 500 votes is likely to have lower variance than averaging over 5 votes.

The construction of the CAWPE ensemble involves estimating the classification accuracy of each base classifier on the train data through a ten-fold cross-validation, then constructing a model of each base classifier on the whole train data. Classifying a new case, described in Algorithm 1 and Eq. 1, requires obtaining a probability estimate of each class from all the base classifiers, weighting these by the cross-validation accuracy raised to the power $\alpha$ (the only parameter of the approach), then either normalising if probability estimates are required or returning the index of the maximum probability if a prediction is needed.
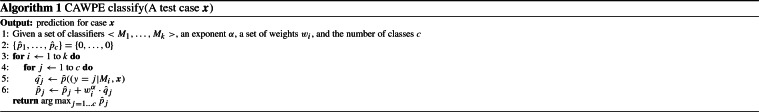


As $\alpha$ increases, the weightings of classifiers found to be stronger on the training data relative to the rest are increased, until the ensemble becomes functionally identical to the single best classifier in training. Conversely, when alpha is 0 all members will be equally weighted. Therefore, on a high level, the $\alpha$ parameter defines the degree to which the base classifiers’ error estimates should be trusted in guiding the ensemble’s output. Set $\alpha$ too high, and all but the best classifier’s outputs are diminished. Set $\alpha$ too low, and the competitive advantage that the best individual is estimating it has is potentially wasted. The quality of the error estimate is key to this process, of course, thus the use of cross-validation as opposed to a single validation set as used in a number of previous works (Kohavi 1995).

The optimal value of $\alpha$ will therefore allow the strongest classifiers to steer the ensemble, but enable them to be overruled when sufficiently outvoted. This value will be dependent on the relative performances and distribution of probabilistic outputs of the base classifiers on the given dataset. To keep in line with the general ethos of simplicity, we remove the need to tune $\alpha$ and potentially overfit it by fixing $\alpha$ to 4 for all experiments and all component structures presented. We chose the value 4 fairly arbitrarily as a sensible starting point before running any experiments. In Sect. 6 we revisit the importance of the $\alpha$ parameter and whether it could benefit from tuning, as well other design decisions we have made.

## Experimental design

The UCI dataset archive^1^ is widely used in the machine learning and data mining literature. An extensive evaluation of 179 classifiers on 121 datasets from the UCI archive, including different implementations of notionally the same classifier, was performed by Fernández-Delgado et al. (2014). It is worth mentioning there have been several problems identified with the experimental procedure used in this study (see Wainberg et al. 2016 for a critique). Firstly, some algorithms were tuned, others were used with the built in default parameters, which are often poor. For example, random forest in Weka defaults to 10 trees. Secondly, for some of the tuned algorithms, there was an overlap between validation and test datasets, which will have introduced bias. Thirdly, the data were formatted to contain only real valued attributes, with the categorical attributes in some data sets being naively converted to real values. We retain this formatting in order to maintain consistency with previous research but this may bias against certain types of classifier. Comparisons between heterogeneous ensembles should be entirely unaffected, since they are all built on the same base classifier prediction information. We have no prior belief as to the impact of the formatting on other base classifiers and in order to avoid any suggestion of a priori bias, we use the exact same 121 datasets. A summary of the data is provided in Table 5 in the “Appendix”.

The UCR archive is a continually growing collection of real valued time series classification (TSC) datasets.^2^ A recent study Bagnall et al. (2017) implemented 18 state-of-the-art TSC classifiers within a common framework and evaluated them on 85 datasets in the archive. The best performing algorithm, the collective of transformation-based ensembles (COTE), was a heterogeneous ensemble of strong classifiers. These results were our primary motivation for further exploring heterogeneous ensembles for classification problems in general.

We aim to use this data to test the generality of some of the core results obtained on the UCI archive, serving as an independent collection of data with entirely different characteristics and separate from the problems with the UCI data described previously. A summary of this data is provided in Table 5 in the “Appendix”.

Experiments are conducted by averaging over 30 stratified resamples. Data, results and code can all be found at the accompanying website for this research.^3^ For the UCI data, 50% of the data is taken for training, 50% for testing. Therefore there is no overlap in train or test data as previously observed by Wainberg et al. (2016) and the data can be used in a similar manner to Wainer and Cawley (2017) without introducing bias. The UCR archive provides a default train/test split. We perform resamples using the number of train and test cases defined in these default splits. We always compare classifiers on the same resamples, and these can be exactly reproduced with the published code. Resample creation is deterministic and can be reproduced using the method

Experiments.sampleDataset(directory,datasetName,foldID), or alternatively the initial train/test split and all resampled folds can be downloaded. This means we can compare two classifiers with paired two sample tests, such as Wilcoxon signed-rank test. For comparing two classifiers on multiple datasets we compare either the number of datasets where there is a significant difference over resamples, or we can do a pairwise comparison of the average errors over all resamples. All code is available and open source. The experiments can be reproduced (see class vector_classifiers.CAWPE). In the course of experiments we have generated gigabytes of prediction information and results. These are available in raw format and in summary spreadsheets. For comparing multiple classifiers on multiple datasets, we follow the recommendation of Demšar (2006) and use the Friedmann test to determine if there are any statistically significant differences in the rankings of the classifiers. However, following recent recommendations by Benavoli et al. (2016) and García and Herrera (2008), we have abandoned the Nemenyi post-hoc test originally used by Demšar (2006) to form cliques (groups of classifiers within which there is no significant difference in ranks). Instead, we compare all classifiers with pairwise Wilcoxon signed-rank tests, and form cliques using the Holm correction (which adjusts family-wise error less conservatively than a Bonferonni adjustment).

We assess classifier performance by four statistics of the predictions and the probability estimates. Predictive power is assessed by test set error and balanced test set error. The quality of the probability estimates is measured with the negative log likelihood (NLL). The ability to rank predictions is estimated by the area under the receiver operator characteristic curve (AUC). For problems with two classes, we treat the minority class as a positive outcome. For multiclass problems, we calculate the AUC for each class and weight it by the class frequency in the train data, as recommended by Provost and Domingos (2003).

## Results

We demonstrate the benefits of the CAWPE scheme through a sequence of experiments to address the following questions:

- Does CAWPE improve heterogeneous base classifiers (Sect. 5.1)?
- Is CAWPE better on average than alternative heterogeneous ensemble schemes all using the same base classifiers and error estimates (Sect. 5.2)?
- Is CAWPE better on average than homogeneous ensembles (Sect. 5.3)?
- How does CAWPE compare to tuned versions of classifiers commonly considered state of the art (Sect. 5.4)?
- Do the results generalise to other data (Sect. 5.5)?

Throughout, we make the associated point that CAWPE is significantly better than its components when they are approximately equivalent. CAWPE has a single parameter, $\alpha$, which is set to the default value of 4 for all experiments. We stress that we perform no tuning of CAWPE’s parameter $\alpha$: it simply combines classifier output using the algorithm described in Algorithm 1. We investigate the sensitivity of CAWPE to $\alpha$ in Sect. 6.3.

We present results in this section through critical difference diagrams which display average rankings. A full list of the average scores for each classifier is provided in Table 6 in the “Appendix”, while further spreadsheets are available on the accompanying website.

### Does CAWPE improve heterogeneous base classifiers?

Ensembling multiple classifiers inherently involves more work than using any single one of them. As a basic sanity check, we assess whether applying CAWPE to a random set of classifiers improves performance. We randomly sampled 5 out of 22 classifiers available in Weka and constructed CAWPE on top of them. Over 200 random configurations, CAWPE was significantly more accurate than the individual component with the best average rank on 143 (71.5%), and insignificantly more accurate on a further 34 (17%), over the 121 UCI datasets. CAWPE was never significantly worse than the best individual component. Note that many of these sets contain components that are significantly different, with average accuracies across the archive ranging between 81.4 and 62.7%.

To avoid confusion as to the components of any CAWPE instantiation, we continue the evaluation with two sets of base classifiers. The first, simpler set contains well known classifiers that are fast to build. These are: logistic regression (Logistic); C4.5 decision tree (C4.5); linear support vector machine (SVML); nearest neighbour classifier (NN); and a multilayer perceptron with a single hidden layer (MLP1). These classifiers are each distinct in their method of modelling the data, and are roughly equivalent in performance. We call this version CAWPE-S.

The second set of five classifiers are more complex, and generally considered more accurate than the previous set. These are: random forest (RandF); rotation forest (RotF); a quadratic support vector machine (SVMQ); a multi layer perceptron implementation with two hidden layers (MLP2); and extreme gradient boosting (XGBoost). We call CAWPE built on this second set of advanced classifiers CAWPE-A.

**Fig. 2:**
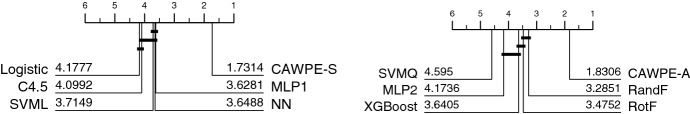
Critical difference diagrams CAWPE-S with its base classifiers (left), and CAWPE-A with its base classifiers (right). Ranks formed on test set accuracy averaged over 30 resamples

In Fig. 2 we compare CAWPE-A and CAWPE-S against their respective base classifiers in terms of accuracy. In both cases, CAWPE is significantly better than all components. CAWPE also significantly improves of all the base components in terms of balanced accuracy, AUROC, and log likelihood.

The improvement is not particularly surprising for CAWPE-S, since the benefits of ensembling weaker learners are well known. It is perhaps more noteworthy, however, that learners often considered state-of-the-art such as random forest, rotation forest and XGBoost, are improved by inclusion in the CAWPE-A ensemble. This improvement is achieved at a computational cost. The CAWPE scheme will require more computation than using a single classifier, since a cross-validation procedure is required for each base classifier. If a ten-fold cross-validation is used, as we do in all our experiments, CAWPE requires approximately 50 times longer to train than the average training time of its five base classifiers. In terms of time taken to predict a new test case, CAWPE simply needs five times the average prediction time of the base classifiers. We have experimentally verified this is the case, but exclude results for brevity (see the associated webpage). This constant time overhead is easy to mitigate against: it is trivial to distribute CAWPE’s base classifiers and even the cross-validation for each classifier can easily be parallelised.

### Is CAWPE better on average than alternative heterogeneous ensemble schemes?

We compare the particular weighting scheme used in CAWPE to well known alternatives. We compare CAWPE-S and CAWPE-A to the weighting, selection and stacking approaches described in Sect. 2. In each comparison, all ensembles use the same set of base classifiers, so the only source of variation is the ensemble scheme. Algorithms such as ensemble selection were originally described as using a single validation set to assess models. However, cross-validation will on average give a better estimate of the true error than a single hold-out validation set (Kohavi 1995). Given that CAWPE uses cross-validation error estimates and that these estimates are already available to us, we also use these for all ensembles. Hence, we are purely testing the ability of the ensembles to combine predictions with exactly the same meta-information available.

**Fig. 3:**
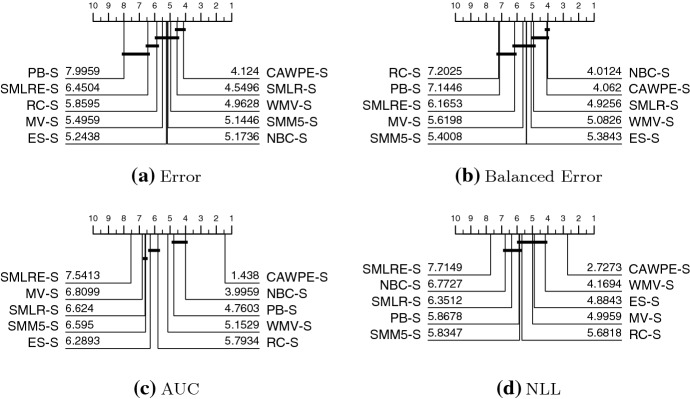
Critical difference diagrams for ten heterogeneous ensemble classifiers on 121 UCI data built using logistic, C4.5, SVML, NN and MLP1 base classifiers. The weighted ensembles are: majority vote (MV); weighted majority vote (WMV); recall (RC); Naive Bayes (NBC) and our scheme (CAWPE). The selection ensembles are: pick best (PB); and ensemble selection (ES). The stacking schemes are: stacking with multi-response linear regression (SMLR); stacking with multi-response linear regression on extended features (SMLRE); and stacking with multi-response model trees (SMM5)

Figure 3 shows the summary ranks of ten heterogeneous ensembles built on the simpler classifier set on the 121 UCI datasets using four performance metrics. CAWPE-S is highest ranked for error and in the top clique for both error and balanced error. It is significantly better than all other approaches for AUC and NLL. It has significantly lower error than all but SMLR, and significantly lower balanced error than all but NBC.

**Fig. 4:**
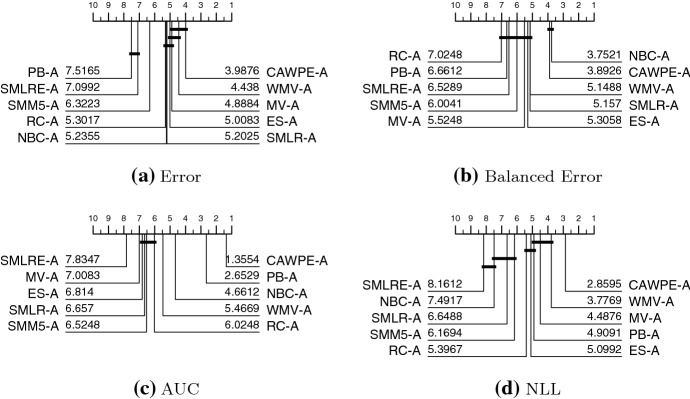
Critical difference diagrams for ten heterogeneous ensemble classifiers on 121 UCI data built using random forest (RandF), rotation forest (RotF), support vector machine with a quadratic kernel (SVMQ), a two layer multilayer perceptron (MLP2) and extreme gradient boosting (XGBoost) base classifiers

Figure 4 shows the summary ranks of the same ten heterogeneous ensembles on the 121 UCI datasets using the more advanced classifiers. The pattern of results is very similar to those for the simple classifiers. CAWPE-A is top ranked for error and in a clique with majority vote and weighted majority vote. For balanced error, it is not significantly different to NBC and is significantly better than the others. For both AUC and NLL, it is significantly better than all the other methods. Considering the results for both CAWPE-S and CAWPE-A, it is apparent that the CAWPE scheme is more consistent than other approaches, since it is the only algorithm in the top clique for all measures for both sets of classifiers. We think this suggests that the CAWPE scheme on this data is the best heterogeneous ensemble technique, at least for the simple and advanced component sets studied.

Given the ensembles are using the same base classifiers and accompanying error estimates, and these are all good classifiers in their own right, we would expect the actual differences in average error to be small, and this is indeed the case (see Table 6 in “Appendix”). Nevertheless, the weighting scheme used in CAWPE is significantly better than nearly all the other methods using the four metrics.

In conclusion, CAWPE makes sets of approximately equivalent classifiers significantly better, and is competitive with or generally better than commonly used weighting, selection and stacking schemes when the number of classifiers is small. Given how simple CAWPE is, we believe it is a sensible starting point for any attempt at combining small numbers of base classifiers on an arbitrary problem. The question then is, should you heterogeneously ensemble at all, or rather should you focus efforts into improving a single model?

### Is CAWPE better on average than homogeneous ensembles?

We examine how CAWPE-S compares to five homogeneous ensembles that each employ 500 duplicates of the same base classifier. CAWPE-A, which includes RandF and XGBoost in its base classifier set, is significantly better on all four performance metrics than both them and all the homogeneous ensembles evaluated here (see Fig. 2, the results are available on the accompanying website). However, this improvement requires roughly 50 times the computational effort of XGBoost or Random Forest alone. We are more interested in assessing how the simpler and faster CAWPE-S compares with homogeneous ensembles.

**Fig. 5:**
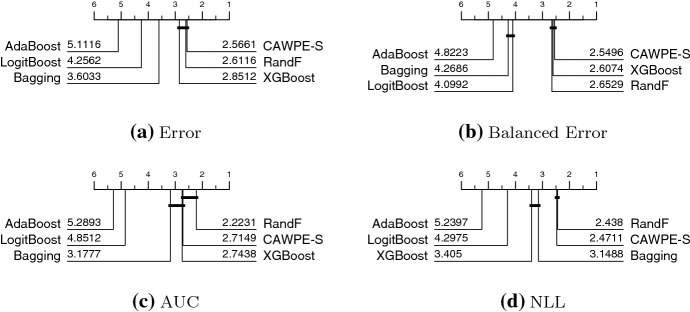
Critical difference diagrams for CAWPE (built using logistic, C4.5, SVML, NN and MLP1 base classifiers) against 5 homogeneous ensemble classifiers on 121 UCI data

Figure 5 shows the results of five ensembles each with 500 base classifiers and CAWPE-S. We observe that CAWPE-S is significantly more accurate than AdaBoost, LogitBoost and Bagging, and not significantly worse than Random Forest and XGBoost. With minimal effort using standard classifiers we have produced an ensemble that is not significantly worse than state-of-the-art homogeneous ensembles.

**Table 1:** Summaries of train times for CAWPE-S and the homogeneous ensembles

Classifier	CAWPE-S	LogitBoost	RandF	XGBoost	Bagging	AdaBoost
Mean	524.9	302.2	111.9	46.8	22.7	7.8
Median	13.7	8.9	6.9	2.1	0.7	0.06

All times are in seconds, and are averaged across the 121 UCI data

Table 1 summarises the train times of CAWPE-S and the homogeneous ensembles in seconds. CAWPE on this simpler component set has a much larger mean train time than RandF and XGBoost. This largely comes down to the logistic regression component, which takes a relatively much longer amount of time on datasets with larger numbers of classes. The median times are closer, however XGBoost especially still achieves predictive performance not significantly different to that of CAWPE-S in much shorter times on average.

These timings should be interpreted with the understanding that XGBoost is a highly optimised library, while the logistic and MLP1 implementations in particular are relatively straight forward and unoptimised implementations in Java. The fact that CAWPE-S has a median train time within the same order of magnitude as XGBoost while not being significantly less accurate is, we think, a positive result.

### How does CAWPE compare to tuned classifiers?

In Sect. 5.1 we showed the ensemble scheme outperforms its set of base classifiers. However, finding the weights requires an order of magnitude more work than building a single classifier because of the ten fold cross-validation across the different components. Given it is widely accepted that tuning parameters on the train data can significantly improve classifier accuracy (Bagnall and Cawley 2017), perhaps a carefully tuned classifier will do as well as or better than CAWPE built on untuned classifiers. To investigate whether this is the case, we tune an SVM with a radial basis function kernel (SVMRBF), XGBoost, MLP and a random forest and compare the results to CAWPE-S and CAWPE-A. We tune by performing a ten-fold cross-validation on each train resample for a large number of possible parameter values, described in Table 2. This requires a huge computational effort. We can distribute resamples and parameter combinations over a reasonably sized cluster. Even so, considerable computation is required; we were unable to complete a full parameter search for 4 datasets (within a 7 day limit): adult; chess-kvrk; miniboone; and magic. To avoid bias, we perform this analysis without these results.

**Table 2:** Tuning parameter ranges for SVMRBF, random forest, MLP and XGBoost

Classifier	Total	Parameter	Range
SVMRBF	1089	Regularisation *C* (33 values)	$\{2^{-16},2^{-15},\ldots ,2^{16}\}$
		Variance $\gamma$ (33 values)	$\{2^{-16},2^{-15},\ldots ,2^{16}\}$
Random forest	1000	Number of trees (10 values)	$\{10,100,200,\ldots ,900\}$
		Feature subset size (10 values)	$\{\sqrt{m},(\log _2{m}+1),\frac{m}{10},\ldots ,\frac{m}{3}\}$
		Max tree depth (10 values)	$\{0,\frac{m}{9},\frac{m}{8}\ldots ,m\}$
MLP	1024	Hidden layers (2 values)	$\{1,2\}$
		Nodes per layer (4 values)	$\{c,m,m+c,\frac{(m+c)}{2}\}$
		Learning rate (8 values)	$\{1,\frac{1}{2},\frac{1}{4},\ldots ,1/(2^7)\}$
		Momentum (8 values)	$\{0,\frac{1}{8},\frac{2}{8},\ldots ,\frac{7}{8}\}$
		Decay (2 values)	$\{true,false\}$
XGBoost	625	Number of trees (5 values)	$\{ 50, 100, 250, 500, 1000 \}$
		Learning rate (5 values)	$\{0.01, 0.05, 0.1, 0.2, 0.3\}$
		Max tree depth (5 values)	$\{ 2,4,6,8,10 \}$
		Min child weight (5 values)	$\{ 1,3,5,7,9 \}$

*c* is the number of classes and *m* the number of attributes

**Fig. 6:**
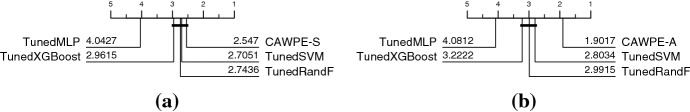
Average ranked errors for **a** CAWPE-S and **b** CAWPE-A against four tuned classifiers on 117 datasets in the UCI archive. The datasets adult, chess-krvk, miniboone and magic are omitted due to computational restraints

Figure 6 compares CAWPE-S and CAWPE-A to tuned versions of MLP, XGBoost, RandF and SVM. On average, CAWPE-S, containing the five simpler untuned base classifiers (Logistic, C4.5, SVML, NN and MLP1), is significantly better than the tuned MLP and not significantly worse than tuned versions of XGBoost, SVMRBF and Random Forest (Fig. 6a). The highest ranked tuned classifier is SVM, but it is still ranked lower than CAWPE-S. This despite the fact that CAWPE-S is two orders of magnitude faster than the tuned SVM and at least one order of magnitude faster than tuned Random Forest, MLP and XGBoost. Sequential execution of CAWPE-S for miniboone (including all internal cross-validation to find the weights) is 5 hours. For TunedSVM, ten-fold cross-validation on 1089 different parameter combinations gives 10,890 models trained for each resample of each dataset. For the slowest dataset (miniboone), sequential execution would have taken more than 6 months. Of course, such extensive tuning may not be necessary. However, the amount and exact method of tuning to perform is in itself very hard to determine. Our observation is that using simple approach such as CAWPE-S avoids the problem of guessing how much to tune completely.

If we use CAWPE-A, containing the more advanced components (RandF, RotF, SVMQ, MLP2 and XGBoost), we get a classifier that is significantly more accurate than any of the individuals (Fig. 6b). CAWPE-A takes significantly longer to train than CAWPE-S, but it is still not slower on average than the tuned classifiers. We are not claiming that CAWPE-A is significantly faster than tuning a base classifier in the general case, because this is obviously dependent on the tuning strategy. CAWPE-A involves a ten fold cross-validation of five classifiers, so it is going to be comparable in run time to one of these single classifiers tuned over 50 parameter settings. However, our experiments demonstrate that tuning a single base learner over a much larger parameter space does not result in as strong of a model, on average.

Our goal is not to propose a particular set of classifiers that should be used with CAWPE. Rather, we maintain that if one has some set of classifiers they wish to apply to problem, ensembling them using CAWPE is generally at least as strong as other heterogeneous ensemble schemes when we have a relatively small number of base classifiers, that it significantly improves base classifiers that are approximately equally strong, and that the degree of improvement is such that state-of-the-art level results can be achieved with minimal effort. Once a classifier is trained and the results are stored, ensembling is very quick. To perhaps belabour the point, we ensembled the four tuned classifiers using the parameter ranges given in Table 2 and the resulting classifier was significantly better than the components in a manner reflecting the patterns observed in Sect. 5.1.

### Does the CAWPE performance generalise to other datasets?

Our interest in heterogeneous ensembles originated in time series classification (TSC) problems, where we ensemble over different representations of the data in a style similar to CAWPE (Lines et al. 2016). TSC involves problems where the attributes are ordered (not necessarily in time) and all real valued. The UCR repository for TSC contains problems from a wide range of domains such as classifying image outlines, EEG and spectrographs. There are currently 85 datasets, with diverse data characteristics. A full list of the 85 datasets is listed in the “Appendix” in Table 5.

Traditionally, dynamic time warping distance (with window size set through cross-validation) (Ratanamahatana and Keogh 2005) with a 1-nearest neighbour classifier (referred to as just DTW henceforth) has been considered the benchmark algorithm for this type of problem. In recent years, a range of bespoke algorithms have been proposed in high impact journals and conferences. The experimental evaluation in Bagnall et al. (2017) found that of 18 such algorithms, only 13 were significantly better (in terms of accuracy) than DTW.

Our goal is to test how well the results observed for CAWPE on the UCI data generalise to other data, by testing whether CAWPE significantly improves over its components on the UCR archive also. To do so, we ignore the ordering of the series and treat each time step in the series as a feature for traditional vector-based classification. The UCR datasets generally have many more features than the UCI data. This has meant we have had to make one change to CAWPE-S: we remove logistic regression because it cannot feasibly be built on many of the data. Since DTW is a 1-nearest neighbour classifier, it always produces 0/1 probability estimates. Because of this, we omit a probabilistic evaluation using AUC and NLL, as it has little meaning for DTW.

**Fig. 7:**
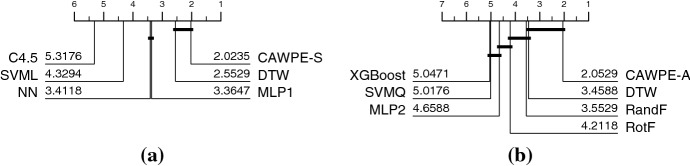
Average ranked errors for DTW against **a** CAWPE-S and its components and **b** CAWPE-A and its components on the 85 datasets in the UCR archive

Figure 7 shows the critical difference diagrams for accuracy of CAWPE-S, CAWPE-A, their respective constituents, and DTW. Both sets of base classifiers are significantly improved by CAWPE once more. These results closely mirror those on the UCI datasets presented above. Furthermore, neither of the CAWPE versions are significantly worse than DTW and both have higher average rank. This should be considered in the context that neither classifier takes advantage of any information in the ordering of attributes. Despite this, CAWPE-A has a higher average rank than 9 of the 18 bespoke time series classification algorithms evaluated in Bagnall et al. (2017), and is not significantly worse than 11 of them. CAWPE, a simple ensemble using off the shelf components and a simple weighting scheme, has been made as accurate as complex algorithms that use a range of complicated techniques such as forming bags of patterns, using edit distance based similarity, differential based distances, compression techniques and decision trees based on short subseries features.

Using standard classifiers for TSC is unlikely to be the best approach. The best performing TSC algorithm in Bagnall et al. (2017), significantly more accurate than all the others, was the Collective of Transformation-based Ensembles (COTE) (Bagnall et al. 2015). It has components built on different representations of the data. COTE uses an ensemble structure that is the progenitor of CAWPE. The latest version of COTE, HIVE-COTE (Lines et al. 2016) uses weighted majority voting for five modularised classifier components defined on shapelet, elastic distance, power spectrum, bag-of-words and interval based representations, and is significantly more accurate than the previous version, flat-COTE, and all of the competing algorithms. HIVE-COTE exploits the diversity of the representations through an ensemble scheme. We address the question of whether CAWPE is the best ensemble scheme for HIVE-COTE.

**Fig. 8:**
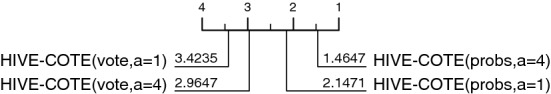
Average ranked errors for 4 variants of HIVE-COTE on the UCR datasets

Figure 8 shows how HIVE-COTE performs when we incrementally add in the CAWPE combination scheme methods. The left most version, weighted majority vote, is the classifier used in Lines et al. (2016). Raising the weight to the power of four significantly reduces error. Switching to using probabilities is significantly better than either weighted voting scheme. Using CAWPE (probs, $\hbox {a}=4$ in Fig. 8) is significantly better than all variants. It is not just a matter of tiny improvements in accuracy improving the ranks. The overall mean accuracy over all problems for HIVE-COTE using CAWPE is 87.16%, whereas the accuracy reported in Lines et al. (2016) using WMV is 85.97%. An overall improvement of over 1% for such a simple change is hugely valuable. For context, the average accuracy of DTW is 77.7%.

## Analysis

We perform a more in-depth analysis of results to determine whether there are any patterns in the results that indicate when and why CAWPE performs well. We compare various facets of performance against choosing the best component on any given dataset (Sect. 6.1). We then perform an ablative study of CAWPE (Sect. 6.2), and a sensitivity study of its parameter, $\alpha$ (Sect. 6.3).

### CAWPE versus pick best exploratory analysis

**Fig. 9:**
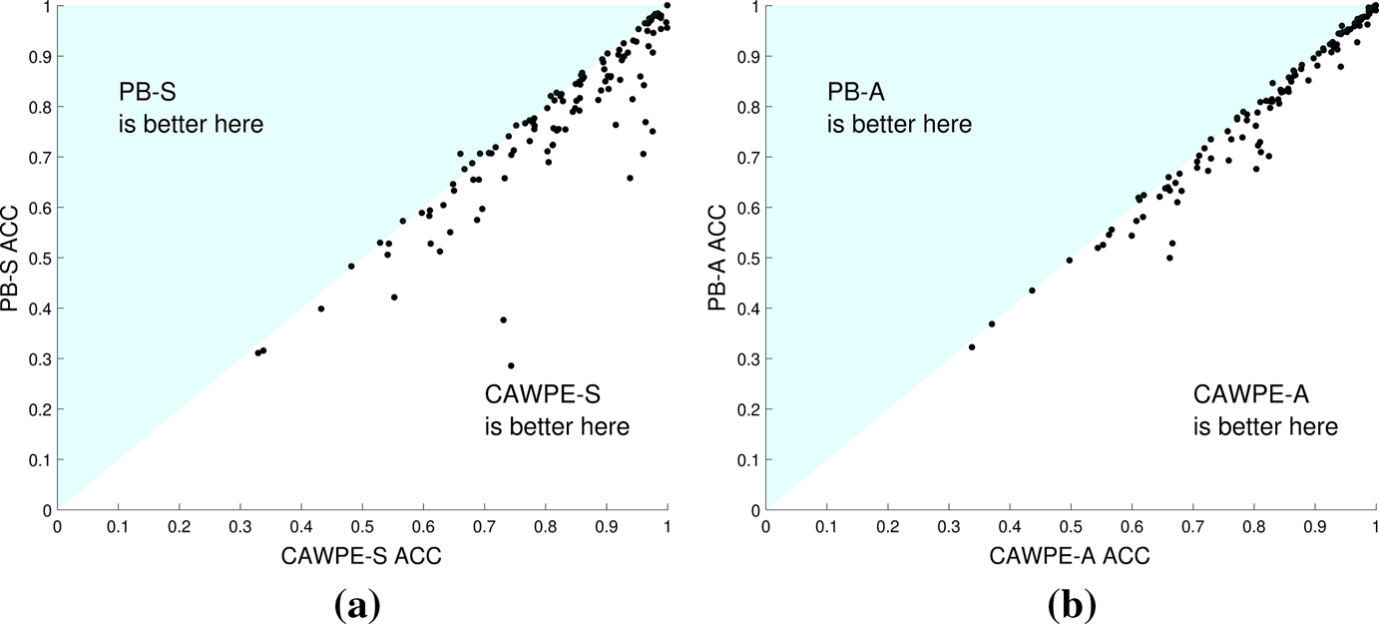
Accuracy of **a** CAWPE-S and **b** CAWPE-A versus picking the best component

Given CAWPE ensembles based on estimates of accuracy obtained from the train data and gives increasingly larger weights to the better classifiers, it seems reasonable to ask, why not just choose the single classifier with the highest estimate of accuracy? Figure 3 demonstrated that it is on average significantly worse choosing a single classifier than using the CAWPE ensembles. When comparing algorithms over entire archives, we get a good sense of those which are better for general purpose classification. However, differences in aggregated ranks do not tell the whole story of differences between classifiers. It could be the case that CAWPE is just more consistent that its components: it could be a jack of all trades ensemble that achieves a high ranking most of the time, but is usually beaten by one or more of its components. A more interesting improvement is an ensemble that consistently achieves higher accuracy than all of its components. For this to happen, the act of ensembling needs to not only cover for the weaknesses of the classifiers when in their suboptimal domains, but accentuate their strengths when within their specialisation too. Figure 9 shows the scatter plots of accuracy for choosing the best base classifier from their respective component sets against using CAWPE. This demonstrates that CAWPE has higher accuracy than Pick Best on the majority of problems, and that the differences are not tiny.

**Fig. 10:**
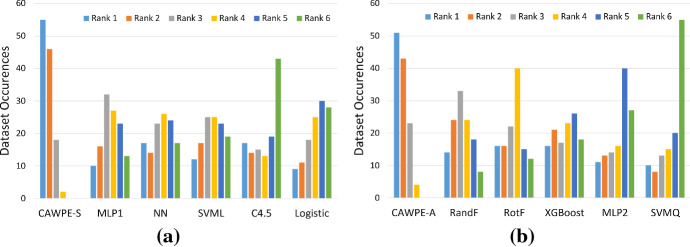
Clustered histograms of accuracy rankings over the 121 UCI datasets for **a** CAWPE-S and **b** CAWPE-A and their respective components. For each classifier, the number of occurrences of each rank being achieved relative to the other classifiers is shown

Figure 10 shows the counts of the rankings achieved by CAWPE built on the simpler (a) and advanced (b) components, in terms of accuracy, over the 121 UCI datasets. CAWPE is the single best classifier far more often than any of its components, and is in fact more often the best classifier than second best. Both versions of CAWPE are never ranked fifth or sixth, and very rarely ranked fourth, demonstrating the consistency of the improvement. This suggests that the simple combination scheme used in CAWPE is able to actively enhance the predictions of its locally specialised members, rather than just achieve a consistently good rank.

For clarity we restrict further analysis to the CAWPE-S results. Comparable results for CAWPE-A are available on the accompanying website.

Comparing overall performance of classifiers is obviously desirable; it addresses the general question: given no other information, what classifier should I use? However, we do have further information. We know the number of train cases, the number of attributes and the number of classes. Does any of this information indicate scenarios where CAWPE is gaining an advantage? The most obvious factor is train set size, since picking the best classifier based on train estimates is likely to be less reliable with small train sets.

**Table 3:** CAWPE-S versus pick best split by train set size

#Train cases	#Problems	#CAWPE-S WINS	Mean error difference (%)
**1–100**	**28**	**21**	**1.49**
**101–500**	**46**	**36**	**0.71**
**501–1000**	**12**	**11**	**1.51**
1001–5000	23	11	0.16
> 5001	9	2	0.02

The three datasets with the same average error have been removed (acute-inflammation, acute-nephritis and breast-cancer-wisc-diag). If there is a significant difference within a group (tested using a Wilcoxon sign rank test) the row is in bold

**Fig. 11:**
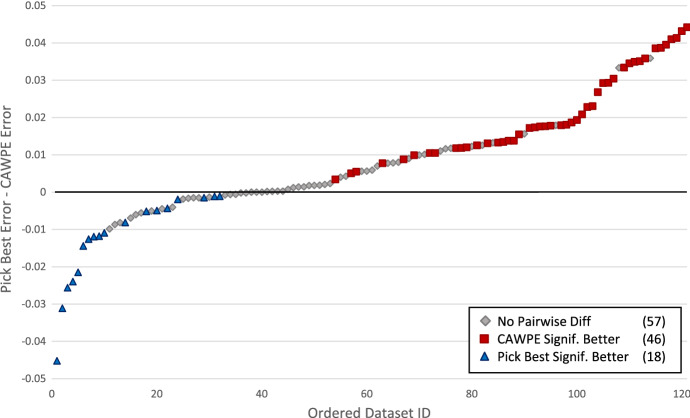
The difference in average errors in increasing order between CAWPE-S and picking the best classifier on each dataset. Significant differences according to paired t-tests over folds are also reported. CAWPE-S is significantly more accurate on 46, the best individual classifier on 18, and there is no significant difference on 57

Table 3 breaks down the results of CAWPE-S compared to Pick Best by train set size. With under 1000 train cases, CAWPE-S is clearly superior. With 1000–5000 cases, there is little difference. With over 5000 cases, CAWPE-S is better on just 2 of 9 problems, but there is only a tiny difference in error. This would indicate that if one has over 5000 cases then there may be little benefit in using CAWPE-S, although it is unlikely to be detrimental. Analysis shows there is no detectable significant effect of number of attributes. For the number of classes, there is a benefit for CAWPE-S on problems with more than 5 classes. CAWPE-S wins on 62% of problems with five or fewer classes (53 out of 85) and wins on 85% of problems with 6 or more (28 out of 33). This is not unexpected, as a large number of classes means fewer cases per class, which is likely to introduce more noise into the estimate of error.

Despite using the same classification algorithms, not all of the differences between pick best and CAWPE-S are small in magnitude. Figure 11 shows the ordered differences between the two approaches. The largest difference in favour of CAWPE-S (averaged over 30 folds) is 4.42% (on the arrhythmia dataset) and in favour of pick best 4.5% (on energy-y1). This demonstrates the importance of the selection method for classifiers; it can cause large differences on unseen data.

This analysis indicates that CAWPE-S is likely to be a better approach than simply picking the best when there is not a large amount of training data, there are a large number of classes and/or the problem is hard. Overall, CAWPE requires almost no extra work beyond pick best and yet is more accurate.

### CAWPE ablative study

CAWPE belongs to the family of ensemble schemes broadly categorised as weighted output combination. We found in Sect. 5 that both CAWPE-S and CAWPE-A are significantly better than the most common instantiations of this type of ensemble; majority vote and weighted majority vote. The major design components of CAWPE are the fact it uses the probabilistic outputs of its base classifiers and the emphasising of differences in weights by using $\alpha$ set to 4. Figure 8 has already shown that that both of these factors result in significant improvement of the TSC algorithm HIVE-COTE. Here we wish to delve further into the contribution that each factor of CAWPE has on its performance. For brevity, we perform all analysis using the CAWPE-S set of simpler classifiers.

**Fig. 12:**
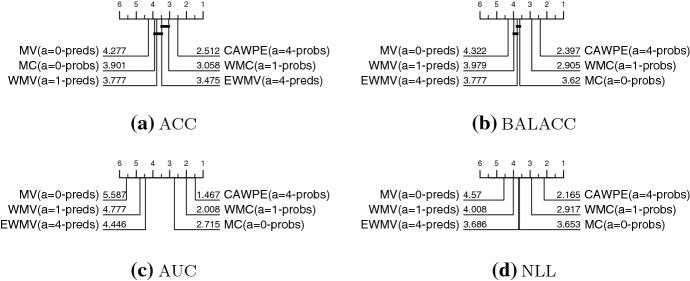
Critical difference diagrams of the stages of progression from a simple majority vote up to CAWPE, on the 121 datasets of the UCI archive using the CAWPE-S variant

**Fig. 13:**
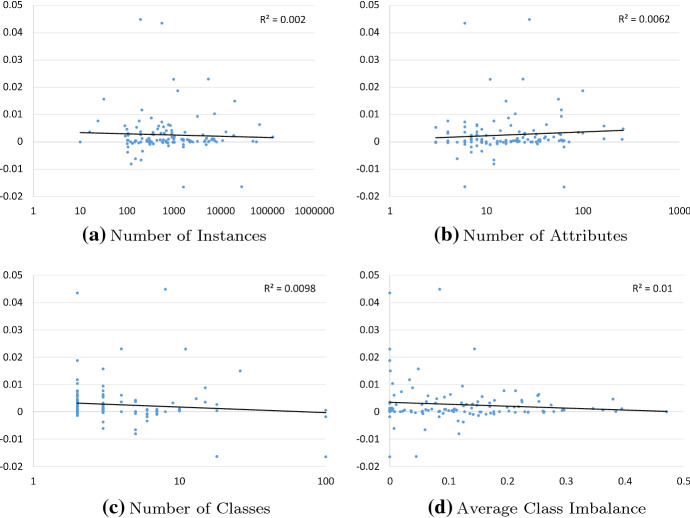
Four plots of the difference in error between CAWPE ($\alpha =4$, probs) and WMC ($\alpha =1$, probs), against different dataset characteristics. Above zero CAWPE wins, below zero WMC wins. Trend represented by solid black line, $R^2$ reported in top-right corner

We split CAWPE based on these two factors, building up from majority vote to CAWPE: the use of the base classifiers’ probabilities (probs) or predictions (preds); and the extent to which we make use of the base classifiers’ cross-validation accuracy to weight their contribution: none at all ($\hbox {a}=0$); standard weighting ($\hbox {a}=1$); and extenuated weighting ($\hbox {a}=4$). Figure 12 details the results of a comparison between all combinations of these factors. To better ground these results in the context of the previous comparison to other heterogeneous ensembles in general in Sect. 5.2, we reuse and define new labels relevant to combinations of these factors of weighted output combination. These are: majority vote (MV: $\hbox {a}=0$, preds); majority confidence (MC: $\hbox {a}=0$, probs); weighted majority vote (WMV: $\hbox {a}=1$, preds); weighted majority confidence (WMC: $\hbox {a}=1$, probs); exponentially weighted majority vote (EWMV: $\hbox {a}=4$, preds); and finally exponentially weighted majority confidence (CAWPE: $\hbox {a}=4$, probs).

These diagrams confirm some suspicions. Firstly, for equal values of $\alpha$, it is always better to use probabilities instead of predictions. For AUC and NLL, the performance metrics most relevant to probabilistic output, the use of probabilities is better even regardless of the value of $\alpha$. Secondly, the use of a weighting scheme, and then further increasing the value of $\alpha$ to 4 also always provides improvement on average.

The improvement from increasing $\alpha$ to 4 is consistent, too, providing in some instances surprising improvements in absolute accuracy. When directly comparing CAWPE ($\alpha =4$, probs) to WMC ($\alpha =1$, probs), CAWPE wins on 86 datasets and loses on 28. The largest reduction in error was 4.49% on the flags dataset, with the largest increase in error being 1.65% on plant-shape.

Figure 13 displays scatter plots to demonstrate these findings. Against differences in error between CAWPE and WMC, it plots a four dataset characteristics: the number of instances; number of attributes; number of classes; and class imbalance. For this purpose, the class imbalance of a dataset is informally calculated as the average difference between each class’ actual proportional representation in the dataset, and its expected value, 1 / *c*. These confirm visually that there is no obvious relationship between the improvement $\alpha$ provides and any of these characteristics.

### CAWPE sensitivity analysis

Section 6.2 has shown that exaggerating the weights of classifiers using $\alpha$ gives a significant increase in performance over standard weighted averaging of probabilities, even with all else being equal. As stated at the end of Sect. 3, the value of $\alpha$ was fixed to 4 for CAWPE for all experiments reported throughout the previous sections. This value was decided on while developing HIVE-COTE. Having performed our experiments with $\alpha =4$, we were interested to find out how sensitive the performance of CAWPE is to this single parameter.

**Fig. 14:**
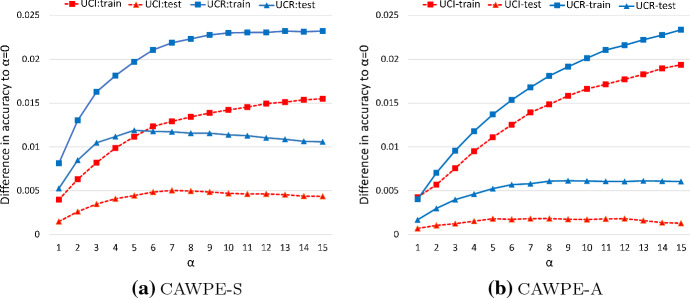
Mean train (squares) and test (triangles) accuracies over the 121 UCI (dashed line) and 85 UCR (solid line) datasets as the alpha parameter changes, expressed as the difference to equal weighting ($\alpha =0$)

Figure 14 depicts what happens if we fix $\alpha$ to progressively higher values over both dataset archives and both base classifier sets used throughout, the basic set (Logistic, C4.5, SVML, NN and MLP1) and the advanced set (RandF, RotF, SVMQ, MLP2 and XGBoost). To keep everything on the same scale and to appropriately highlight the actual differences in accuracy, the average accuracy of each $\alpha$ value is expressed as the difference between itself and using $\alpha =0$, i.e. no weighting of the base classifiers. Even across the two different archives and base classifier sets, the test performances of different values of $\alpha$ show a fairly consistent pattern, rising steadily until around five to seven before tapering off or eventually falling again. Ultimately as $\alpha$ tends to infinity, we know that the ensemble becomes equivalent to picking the best individual, at which point the line has fallen far below 0 on these graphs. While not included for the sake of space and clarity, the results for the other three test statistics (balanced error, AUC, and NLL) follow an effectively identical pattern.

These results give us an understanding of the surprisingly consistent properties of $\alpha$ overall. However, given some particular set of base classifiers, their relative performances and ability to estimate their own performance on the training set could vary to different extents depending on the individual dataset provided. As such, the amount that we want to extenuate the differences between the classifier could change from dataset to dataset. It is therefore natural to wonder whether the alpha parameter could be tuned. To do this in a completely fair and unbiased way, we would need to perform a further nested level of cross-validation. However, we can find a much faster (but possibly biased) estimate of the ensemble’s error by using exactly the same folds as the base classifiers once more, and simply recombining their predictions.

**Fig. 15:**
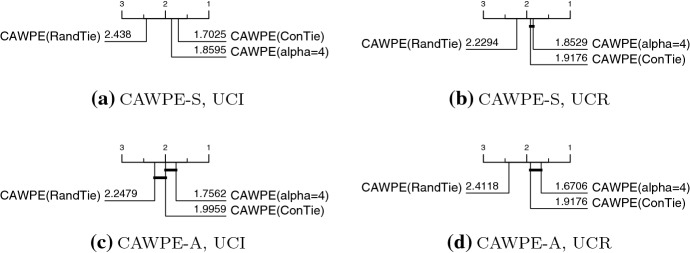
Critical difference diagrams over test error of CAWPE on the UCI and UCR archives as it stands (alpha $=4$), and against two tuning schemes for the alpha parameter: resolving ties in error estimates randomly (RandTie); and conservatively picking the lowest alpha amongst the ties (ConTie)

However, as Fig. 15 shows, tuning alpha over the range $\{0,1,\ldots 15,\infty \}$ appears to offer little to no benefit when doing so with simple and sensible tuning rules such as picking the $\alpha$ with the best accuracy estimate, and resolving ties (which can be quite common in this scenario) either randomly (RandTie) or conservatively, by choosing the smallest tied value of $\alpha$ (ConTie). ConTie tends towards more evenly averaging the base classifier’s outputs, both to counteract any potential overfitting by the base classifiers and, as shown in Fig. 14, the tendency for higher values of $\alpha$ to increasingly lead to higher estimates of the ensemble’s own performance incorrectly.

One could imagine many more complex tuning schemes potentially having a positive effect, such as sticking to the default value of 4, and only deviating if another value significantly improves accuracy over the cross-validation folds. However, considering both this analysis of $\alpha$ and the findings of the previous section, and remembering our initial guiding principle of simplicity, we believe we can reasonably fall back to fixing the value of $\alpha$.

## Conclusions

The key message of this paper is simple: forming heterogeneous ensembles of approximately equivalent classifiers produces on average a significantly better classifier (in terms of error, ordering and probability estimates) than a wide range of potential base classifiers, and that when we use a weighted probabilistic combination mechanism, ensembles of simple classifier can be at least as good as homogeneous ensembles, heterogeneous ensembles or tuned classifiers. The CAWPE method we propose is significantly better than many equivalent methods and, if the number of classifiers being ensembled is relatively small, represents a sensible starting point. CAWPE is quick, simple and easy to understand. The CAWPE of five simple untuned classifiers is not significantly worse than heavily tuned support vector machines, multilayer perceptron, random forest and XGBoost. CAWPE is significantly better than similar heterogeneous schemes based on predictions rather than probabilities. Clearly, CAWPE is not always the best approach, but given the short time it takes to build the simple classifiers we have used to test it, it seems a sensible starting point.

CAWPE has limitations or areas where it is untested. Firstly, as the train set size increases, the value in ensembling, as opposed to just picking the best, reduces. However, picking best rather than ensembling requires a similar amount of work, and ensembling is unlikely to make things worse. Secondly, with a larger pool of classifiers, it may be better to select a subset rather than use all classifiers using some ES type algorithm. We have not tested this, because unless we choose the overproduce and select methodology of including multiple copies of the same learning algorithm, there are not that many learning algorithms that would be considered equivalent. Our approach is to use fewer very different base classifiers, then combine their output in a way that retains the maximum information. Thirdly, it may well be possible that advanced classifiers such as boosting, deep learning and support vector machines can be designed to beat CAWPE, but if this is the case it is not trivial, as we have shown. Finally, the data we have used has only continuous attributes. We made this decision based on the fact that we wanted to extend previous research and because we come to this problem from time series classification, where all data is real valued. It may be that the variation in classifier performance on nominal data is such that the ensembling does not benefit. However, given that CAWPE is classifier neutral, it seems unlikely that the pattern of results would be much different.

Ultimately we hope to drive a better understanding of what classifier to use for a new problem and how best to use it. With current technology, our conclusion is that, rather than expend extra computational time tuning a single classifier, it is better to ensemble different classifiers from different families of algorithms, and that the best way of doing this is to weight the probability estimates from each base classifier with an exponentiated accuracy estimate derived from the train data.

## Appendix

See Tables 4, 5 and 6.

**Table 4:** A full list of the UCI datasets used in the experiments in Sects. 5.1–5.4

Dataset	Atts	Classes	Cases	Dataset	Atts	Classes	Cases
abalone	8	3	4177	monks-1	6	2	556
acute-inflammation	6	2	120	monks-2	6	2	601
acute-nephritis	6	2	120	monks-3	6	2	554
adult	14	2	48,842	mushroom	21	2	8124
annealing	31	5	898	musk-1	166	2	476
arrhythmia	262	13	452	musk-2	166	2	6598
audiology-std	59	18	196	nursery	8	5	12,960
balance-scale	4	3	625	oocytes_m_nucleus_4d	41	2	1022
balloons	4	2	16	oocytes_m_states_2f	25	3	1022
bank	16	2	4521	oocytes_t_nucleus_2f	25	2	912
blood	4	2	748	oocytes_t_states_5b	32	3	912
breast-cancer	9	2	286	optical	62	10	5620
breast-cancer-w	9	2	699	ozone	72	2	2536
breast-cancer-w-diag	30	2	569	page-blocks	10	5	5473
breast-cancer-w-prog	33	2	198	parkinsons	22	2	195
breast-tissue	9	6	106	pendigits	16	10	10,992
car	6	4	1728	pima	8	2	768
cardio-10clases	21	10	2126	pit-bri-MATERIAL	7	3	106
cardio-3clases	21	3	2126	pit-bri-REL-L	7	3	103
chess-krvk	6	18	28,056	pit-bri-SPAN	7	3	92
chess-krvkp	36	2	3196	pit-bri-T-OR-D	7	2	102
congressional-voting	16	2	435	pit-bridges-TYPE	7	6	105
conn-bench-sonar$\ldots$	60	2	208	planning	12	2	182
conn-bench-vowel$\ldots$	11	11	990	plant-margin	64	100	1600
connect-4	42	2	67,557	plant-shape	64	100	1600
contrac	9	3	1473	plant-texture	64	100	1599
credit-approval	15	2	690	post-operative	8	3	90
cylinder-bands	35	2	512	primary-tumor	17	15	330
dermatology	34	6	366	ringnorm	20	2	7400
echocardiogram	10	2	131	seeds	7	3	210
ecoli	7	8	336	semeion	256	10	1593
energy-y1	8	3	768	soybean	35	18	683
energy-y2	8	3	768	spambase	57	2	4601
fertility	9	2	100	spect	22	2	265
flags	28	8	194	spectf	44	2	267
glass	9	6	214	statlog-aus-credit	14	2	690
haberman-survival	3	2	306	statlog-ger-credit	24	2	1000
hayes-roth	3	3	160	statlog-heart	13	2	270
heart-cleveland	13	5	303	statlog-image	18	7	2310
heart-hungarian	12	2	294	statlog-landsat	36	6	6435
heart-switzerland	12	5	123	statlog-shuttle	9	7	58,000
heart-va	12	5	200	statlog-vehicle	18	4	846
hepatitis	19	2	155	steel-plates	27	7	1941
hill-valley	100	2	1212	synthetic-control	60	6	600
horse-colic	25	2	368	teaching	5	3	151
ilpd-indian-liver	9	2	583	thyroid	21	3	7200
image-segmentation	18	7	2310	tic-tac-toe	9	2	958
ionosphere	33	2	351	titanic	3	2	2201
iris	4	3	150	trains	29	2	10
led-display	7	10	1000	twonorm	20	2	7400
lenses	4	3	24	vert-col-2clases	6	2	310
letter	16	26	20,000	vert-col-3clases	6	3	310
libras	90	15	360	wall-following	24	4	5456
low-res-spect	100	9	531	waveform	21	3	5000
lung-cancer	56	3	32	waveform-noise	40	3	5000
lymphography	18	4	148	wine	13	3	178
magic	10	2	19,020	wine-quality-red	11	6	1599
mammographic	5	2	961	wine-quality-white	11	7	4898
miniboone	50	2	130,064	yeast	8	10	1484
molec-biol-promoter	57	2	106	zoo	16	7	101
molec-biol-splice	60	3	3190				

Experiments were conducted on 30 stratified resamples of each dataset, with 50% of the data taken for training and 50% for testing. All classifiers were are aligned on the same folds

**Table 5:** The 85 UCR time series classification problems used in the experiments for Sect. 5.5

Dataset	Atts	Classes	Train	Test	Dataset	Atts	Classes	Train	Test
Adiac	176	37	390	391	MedicalImages	99	10	381	760
ArrowHead	251	3	36	175	MidPhalOutAgeGroup	80	3	400	154
Beef	470	5	30	30	MidPhalOutCorrect	80	2	600	291
BeetleFly	512	2	20	20	MiddlePhalanxTW	80	6	399	154
BirdChicken	512	2	20	20	MoteStrain	84	2	20	1252
Car	577	4	60	60	NonInvasiveThorax1	750	42	1800	1965
CBF	128	3	30	900	NonInvasiveThorax2	750	42	1800	1965
ChlorineConcentration	166	3	467	3840	OliveOil	570	4	30	30
CinCECGtorso	1639	4	40	1380	OSULeaf	427	6	200	242
Coffee	286	2	28	28	PhalOutCorrect	80	2	1800	858
Computers	720	2	250	250	Phoneme	1024	39	214	1896
CricketX	300	12	390	390	Plane	144	7	105	105
CricketY	300	12	390	390	ProxPhalOutAgeGroup	80	3	400	205
CricketZ	300	12	390	390	ProxPhalOutCorrect	80	2	600	291
DiatomSizeReduction	345	4	16	306	ProximalPhalanxTW	80	6	400	205
DisPhalOutAgeGroup	80	3	400	139	RefrigerationDevices	720	3	375	375
DisPhalOutCor	80	2	600	276	ScreenType	720	3	375	375
DislPhalTW	80	6	400	139	ShapeletSim	500	2	20	180
Earthquakes	512	2	322	139	ShapesAll	512	60	600	600
ECG200	96	2	100	100	SmallKitchApps	720	3	375	375
ECG5000	140	5	500	4500	SonyAIBORSurface1	70	2	20	601
ECGFiveDays	136	2	23	861	SonyAIBORSurface2	65	2	27	953
ElectricDevices	96	7	8926	7711	StarlightCurves	1024	3	1000	8236
FaceAll	131	14	560	1690	Strawberry	235	2	613	370
FaceFour	350	4	24	88	SwedishLeaf	128	15	500	625
FacesUCR	131	14	200	2050	Symbols	398	6	25	995
FiftyWords	270	50	450	455	SyntheticControl	60	6	300	300
Fish	463	7	175	175	ToeSegmentation1	277	2	40	228
FordA	500	2	3601	1320	ToeSegmentation2	343	2	36	130
FordB	500	2	3636	810	Trace	275	4	100	100
GunPoint	150	2	50	150	TwoLeadECG	82	2	23	1139
Ham	431	2	109	105	TwoPatterns	128	4	1000	4000
HandOutlines	2709	2	1000	370	UWaveAll	945	8	896	3582
Haptics	1092	5	155	308	UWaveX	315	8	896	3582
Herring	512	2	64	64	UWaveY	315	8	896	3582
InlineSkate	1882	7	100	550	UWaveZ	315	8	896	3582
InsectWingbeatSound	256	11	220	1980	Wafer	152	2	1000	6164
ItalyPowerDemand	24	2	67	1029	Wine	234	2	57	54
LargeKitchApps	720	3	375	375	WordSynonyms	270	25	267	638
Lightning2	637	2	60	61	Worms	900	5	181	77
Lightning7	319	7	70	73	WormsTwoClass	900	2	181	77
Mallat	1024	8	55	2345	Yoga	426	2	300	3000
Meat	448	3	60	60					

Experiments were conducted on 30 stratified resamples of each dataset and all classifiers were aligned on the same folds. Each UCR dataset has an initial default train and test partition that was used for the first experiment, and each subsequent experiment was conducted using resamples of the data that preserve the class distributions and size of the original training and test partitions

**Table 6:** Raw average scores for error, balanced error, AUC and NLL of the classifiers referenced throughout Sect. 5

121 UCI datasets	Classifier	Sections	Error	Balanced error	AUC	NLL
CAWPE	CAWPE-A	5.1, 5.2, 5.4	0.174	0.243	0.893	0.651
	CAWPE-S	5.1, 5.2, 5.3, 5.4	0.184	0.258	0.884	0.706
Simple components	C4.5	5.1	0.23	0.301	0.736	1.161
	Logistic	5.1	0.238	0.309	0.841	8.134
	MLP1	5.1	0.213	0.287	0.86	1.297
	NN	5.1	0.216	0.303	0.798	1.116
	SVML	5.1	0.229	0.306	0.849	1.073
Advanced components	MLP2	5.1	0.204	0.276	0.858	1.26
	RandF	5.1, 5.3	0.185	0.259	0.886	0.713
	RotF	5.1	0.187	0.265	0.868	0.704
	XGBoost	5.1, 5.3	0.193	0.261	0.876	0.843
	SVMQ	5.1	0.216	0.281	0.863	1.454
Heterogeneous ensembles,	ES-S	5.2	0.19	0.266	0.813	0.884
simple components	MV-S	5.2	0.195	0.273	0.808	0.877
	NBC-S	5.2	0.193	0.26	0.82	0.999
	PB-S	5.2	0.229	0.306	0.847	0.95
	RC-S	5.2	0.195	0.288	0.811	0.912
	SMLR-S	5.2	0.195	0.272	0.737	1.144
	SMLRE-S	5.2	0.214	0.288	0.734	1.251
	SMM5-S	5.2	0.195	0.271	0.744	1.046
	WMV-S	5.2	0.192	0.27	0.814	0.872
Heterogeneous ensembles,	ES-A	5.2	0.176	0.246	0.817	0.847
advanced components	MV-A	5.2	0.176	0.249	0.815	0.833
	NBC-A	5.2	0.183	0.249	0.821	1.031
	PB-A	5.2	0.193	0.261	0.876	0.843
	RC-A	5.2	0.177	0.262	0.813	0.87
	SMLR-A	5.2	0.19	0.263	0.752	1.141
	SMLRE-A	5.2	0.203	0.275	0.747	1.232
	SMM5-A	5.2	0.188	0.261	0.757	1.019
	WMV-A	5.2	0.175	0.248	0.817	0.837
Homogeneous ensembles	AdaBoost	5.3	0.353	0.469	0.775	3.258
(RandF and XGBoost repeated)	Bagging	5.3	0.206	0.303	0.868	0.775
	LogitBoost	5.3	0.241	0.302	0.836	8.246
	RandF	5.1, 5.3	0.185	0.259	0.886	0.713
	XGBoost	5.1, 5.3	0.193	0.261	0.876	0.843
Tuned classifiers	TunedMLP	5.4	0.227	0.318	0.857	1.009
	TunedRandF	5.4	0.188	0.271	0.879	0.719
	TunedSVM	5.4	0.188	0.255	0.857	0.955
(On 117 UCI datasets)	TunedXGBoost	5.4	0.194	0.267	0.869	0.86
	CAWPE-T	5.4	0.175	0.244	0.891	0.653

85 UCR datasets	Classifier	Sections	Error	Balanced Error	AUC	NLL
CAWPE	CAWPE-S	5.5	0.241	0.267	0.88	1.071
	CAWPE-A	5.5	0.226	0.254	0.903	0.906
DTW	DTW	5.5	0.224	0.246	–	–
Simple components	C4.5	5.5	0.36	0.384	0.685	2.168
	Logistic	5.5	–	–	–	–
	MLP1	5.5	0.275	0.301	0.842	3.323
	NN	5.5	0.27	0.301	0.78	1.654
	SVML	5.5	0.312	0.337	0.823	4.733
Advanced components	MLP2	5.5	0.276	0.304	0.858	1.538
	RandF	5.5	0.245	0.28	0.893	1.036
	RotF	5.5	0.251	0.279	0.881	1.019
	SVMQ	5.5	0.276	0.295	0.856	5.755
	XGBoost	5.5	0.267	0.297	0.881	1.156

Scores are averaged over all datasets and resamples of the UCI and UCR archives respectively, except for the tuned classifiers on the UCI archive which had the adult, chess-kvrk, miniboone, and magic datasets removed due to computational restraints

## Footnotes

1 http://archive.ics.uci.edu/ml/index.php.

2 http://www.timeseriesclassification.com.

3 http://www.timeseriesclassification.com/CAWPE.php.
